# Survival Outcomes in Sinonasal Mucosal Melanoma: Systematic Review and Meta-Analysis

**DOI:** 10.3390/jpm14121120

**Published:** 2024-11-26

**Authors:** María Jesús Rojas-Lechuga, Sara Jubés, Manuel Molina-García, Rui Milton Patricio da Silva-Júnior, Claudio Sampieri, Cristóbal Langdon, Juan Ramón Gras-Cabrerizo, Manuel Bernal-Sprekelsen, Susana Puig, Isam Alobid

**Affiliations:** 1 Otorhinolaryngology Department, Hospital Clinic of Barcelona, Universitat de Barcelona (UB), 08036 Barcelona, Spain; mrojas@clinic.cat (M.J.R.-L.);; 2 Melanoma Unit, Dermatology Department, Hospital Clinic Barcelona, Universitat de Barcelona (UB), 08036 Barcelona, Spainspuig@clinic.cat (S.P.); 3 Institut d’Investigacions Biomèdiques August Pi i Sunyer (IDIBAPS), CIBERES, 08036 Barcelona, Spain; 4 Otorhinolaryngology Department, Hospital Sant Joan de Déu, 08950 Barcelona, Spain; 5 Otorhinolaryngology Head-Neck Surgery Department, Hospital de la Santa Creu i Sant Pau, Universitat Autònoma de Barcelona, 08025 Barcelona, Spain; 6 Centre of Biomedical Research on Rare Diseases (CIBERER), Instituto de Salud Carlos III, 08034 Barcelona, Spain

**Keywords:** sinonasal mucosal melanoma, survival, prognosis, overall survival

## Abstract

**Background/Objectives**: Sinonasal mucosal melanomas (SNMMs) are rare and aggressive malignancies with poor survival outcomes. Our systematic review and meta-analysis aim to evaluate overall survival (OS) rates in patients with SNMM; **Methods**: We conducted a systematic search, following PRISMA guidelines across PubMed, Web of Science (WOS), and citation searching for studies reporting survival and prognosis outcomes for SNMMs. Inclusion criteria included studies with 5-year OS rates. Studies were excluded if they included tumor sites other than the paranasal sinuses or nasal cavity, were published in languages other than English and Spanish, or had a sample size of fewer than 15 patients. Two reviewers independently screened studies, extracted data, and assessed study quality using the Joanna Briggs Institute (JBI) critical appraisal checklist. Analyses of survival probabilities were conducted. Meta-analyses were performed using a random-effects model. PROSPERO ID CRD42024565137; **Results**: A total of 515 articles were identified after removing duplicates, and 99 reports were assessed for eligibility. Of these, 35 studies were included in the meta-analysis, encompassing a total of 2383 SNMM patients, of whom 1192 (50%) were female, with a weighted mean age of 65.4 years (SD = 5.4). Fifteen studies were from Europe (42.9%), six (17.1%) were from America, eleven (31.4%) were from Asia, two (5.7%) were from Australia, and one (2.9%) combined European, United Kingdom, and American populations. The 5-year OS was 34.8 [95% CI = 30.6–39.5], with the highest OS in America at 40.5 [95% CI = 34.1–48.1], followed by Europe at 36.6 [95% CI = 30.6–43.7], Australia at 32.3 [95% CI = 12.5–83.8], and Asia at 28.1 [95% CI = 19.5–40.7]. The age-standardized incidence rate (ASIR) for SNMM ranges was between 0.07 and 0.14 per 100,000 persons/year, with a slightly higher incidence in women than in men; **Conclusions**: This meta-analysis, one of the largest to date on SNMM, confirms the aggressive nature of this melanoma subtype with poor survival outcomes. Despite geographic differences in survival rates, the overall 5-year survival remains low, highlighting the urgent need for improved treatment strategies and more research to improve patient outcomes.

## 1. Introduction

Sinonasal mucosal melanoma (SNMM) is a rare and aggressive form of melanoma that arises in the mucosa of the nasal cavity and paranasal sinuses. First described by *Lücke* in 1869 [1], it accounts for 1–12% of all sinonasal neoplasms [2], less than 2% of all melanomas [3], and about 60–70% of head and neck mucosal melanomas [4].

SNMM arises from melanocytes of neuroectodermal origin [5]. Several molecular players and signaling pathways are involved in the (dys) regulation of the melanocyte lineage from the neural crest to melanoma genesis [6]. However, the etiologic factors driving SNMM have not yet been elucidated [7].

The incidence is between 0.02 and 0.12 per 100,000/persons per year [8,9]. Despite the incidence appearing to be similar between populations, SNMM represents a greater proportion of melanoma cases in non-European descent (Hispanics excluded) [10,11,12,13].

SNMM predominantly affects older adults, with a median age at diagnosis of around 70 years, and it has a slight female predominance [14]. The clinical presentation of SNMM is often nonspecific, leading to delays in diagnosis. Common symptoms include epistaxis, nasal obstruction, and facial pain or swelling, which can be mistaken for benign sinonasal conditions [15]. Diagnostic workup typically involves a combination of endoscopic examination, imaging studies such as computed tomography (CT), and/or magnetic resonance imaging (MRI). Histopathological confirmation through biopsy [16] is usually challenging due to the similar histopathological features with other sinonasal neuroectodermal and neuroendocrine carcinomas, along with soft tissue sarcomas [17,18], who among others can often mimic SNMM. Immunohistochemical analyses are usually positive for at least one melanocytic marker [19].

The rarity of SNMM, combined with its often late-stage diagnosis and aggressive behavior, usually leads to a poor prognosis. This poor survival is reflected in the AJCC’s staging system for mucosal melanomas of the head and neck, starting at stage III and the T-stage, which is limited to T3 and T4 disease [20].

The management of SNMM can be challenging due to its anatomical location and the propensity for local invasion and distant metastasis [21]. Surgical resection with wide margins is the primary treatment modality [22]; however, achieving clear margins is difficult due to the proximity to critical structures and the frequent occurrence of multifocal lesions [23]. Adjuvant radiotherapy and, more recently, proton beam therapy have been employed to improve local control, but their impact on overall survival remains limited [16].

Despite advances in surgical techniques and adjuvant therapies, the prognosis for SNMM remains poor, with the 5-year overall survival rate ranging from 20% to 45% [24]. The most common cause of death is distant metastasis, highlighting the need for effective systemic therapies. Immunotherapy and targeted therapy, which are a routine part of treatment for cutaneous melanoma, are part of the treatment in SNMM, but their efficacy is not yet well established [25].

The following systematic review and meta-analysis aim to examine the available evidence on survival outcomes in SNMM.

## 2. Materials and Methods

This systematic review was performed in accordance with PRISMA guidelines, and a formal PROSPERO protocol was published (No. CRD42024565137).

### 2.1. Eligibility Criteria

This review included studies with patients diagnosed with SNMM. Studies involving other types of sinonasal tumors or mucosal melanomas from different locations were excluded. All forms of intervention were considered. The primary outcome measured was the 5-year overall survival (OS), while secondary outcomes included survival outcomes stratified by tumor staging and geographical area.

### 2.2. Information Sources

A comprehensive literature search was conducted using PubMed and Web of Science. The search strategy included terms specific to SNMM ((sinonasal mucosal melanoma[Title/Abstract]) OR (sinonasal melanoma[Title/Abstract]) OR ((sinonasal[Title/Abstract]) AND (melanoma[Title/Abstract]))) NOT (Review[Publication Type])). Additional sources included citation searching. The search was limited to articles published in English and Spanish and excluded review articles. The search covered all articles published up to 5 August 2024.

### 2.3. Study Selection

Study selection involved a two-step process. Initially, titles and abstracts were screened to exclude articles not in English or Spanish, studies with fewer than 15 patients, studies involving other tumor subsites, studies unrelated to SNMM, non-human studies, and review articles. Articles that passed this initial screening underwent full-text review, where further exclusions were performed for studies lacking sufficient clinical data or those that were superseded by more recent studies with larger sample sizes by the same authors. Original studies were preferred if available over registry databases. The initial screening and full-text review were independently conducted by two researchers, with discrepancies being resolved through discussion.

### 2.4. Data Collection Process

Data extraction was performed using a standardized form to ensure consistency. Extracted data included study characteristics (type of study, unicentric or multicentric, period of study), participant demographics (number of patients, number and percentage of women and men, age), survival outcomes (overall survival at 5 years, median or mean follow-up), tumor staging, primary tumor site, primary and adjuvant treatments, and genetic mutations or prognostic biomarkers of SNMM.

### 2.5. Risk of Bias in Individual Studies and Pooled Evidence

The risk of bias in individual studies was assessed using the Joanna Briggs Institute (JBI) Critical Appraisal Tools for Case Series [26]. Two reviewers independently evaluated the risk of bias, and disagreements were resolved through discussion and consensus. The quality of pooled evidence at the outcome level was evaluated using the Grading of Recommendations Assessment, Development, and Evaluation (GRADE) system, which accounts for evidence inconsistency, indirectness, imprecision, publication bias, and risk of bias [27].

### 2.6. Summary Measures, Synthesis of Results, and Additional Analyses

The primary summary measure used was overall survival. Where appropriate, data from individual studies were pooled using a random-effects meta-analysis to account for variability between studies. For outcomes where meta-analysis was not feasible, a narrative synthesis was conducted. Subgroup analyses were performed to explore tumor staging and geographical area survival outcomes.

### 2.7. Risk of Bias Across Studies

To assess the risk of bias across studies, funnel plots and Egger’s test were utilized to detect potential publication bias.

### 2.8. SNMM Incidence Rate

Additionally, we describe the age-standardized incidence rate (ASIR) of SNMM as reported to date. For those that provided ASIRs divided by sex, a weighted average by sex was calculated to obtain the overall ASIR. When data were not available, estimates were derived from numbers or graphs.

### 2.9. Data Synthesis and Statistical Analysis

Categorical variables were summarized by counts and percentages, while continuous variables were reported as medians and interquartile ranges or means and ranges when available. Clinical measures were reported as provided by the individual studies. I^2^ was calculated as a measure of heterogeneity. A meta-analysis of OS was performed using a random-effect model, with results being presented in a forest plot graph. Statistical significance was set at ɑ = 0.05, and a 95% confidence interval (CI) was described. All statistical analyses were conducted using STATA software v.16.1 (StataCorp, TX, USA).

## 3. Results

### 3.1. Literature Search Results

**Figure 1:**
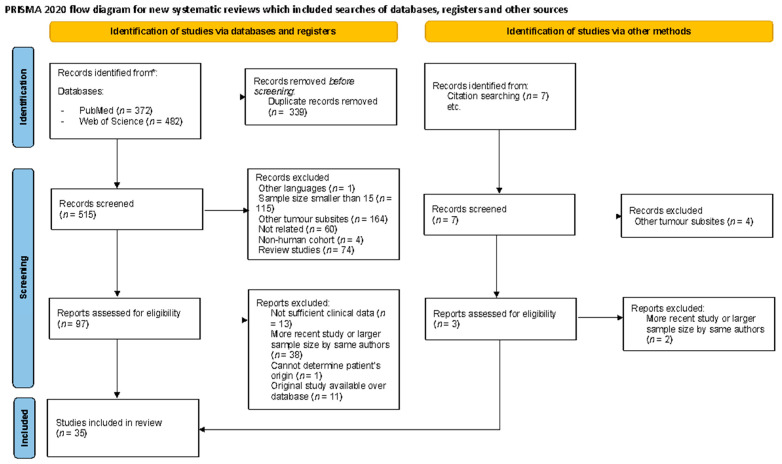
PRISMA flow diagram for systematic review and meta-analysis illustrating study selection [28].

A flowchart of the study identification process is presented in Figure 1. The search strategy retrieved 515 articles after removing duplicates. Following title and abstract screening, 422 articles were excluded, and the full texts of the remaining 99 papers were obtained and reviewed. After applying the inclusion and exclusion criteria, a total of 35 studies were included in both the qualitative and quantitative synthesis (Table 1). The reasons for exclusion are detailed in Supplementary File S1.

### 3.2. Methodological Quality and Risk of Bias of Included Studies

**Figure 2:**
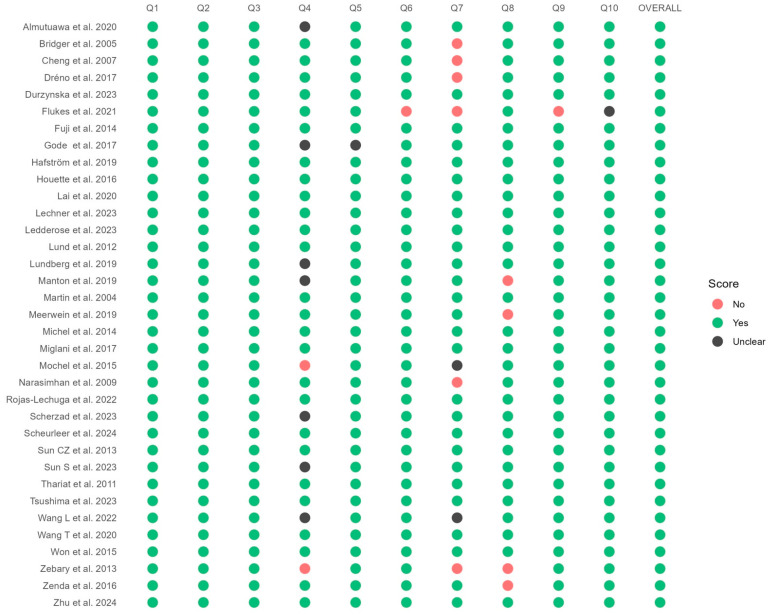
Joanna Briggs Institute Critical Appraisal Tool for risk of bias [9,14,15,26,29,30,31,32,33,34,35,36,37,38,39,40,41,42,43,44,45,46,47,48,49,50,51,52,53,54,55,56,57,58,59,60].

We used the JBI critical appraisal tool to assess the 35 studies, all involving the consecutive inclusion of patients. The studies were moderate to high quality, with a trend toward higher quality in more recent studies (Figure 2). The quality of pooled evidence for overall survival using the GRADE system showed very little confidence in the effect estimate (Supplementary File S2).

### 3.3. Publication Bias

**Figure 3:**
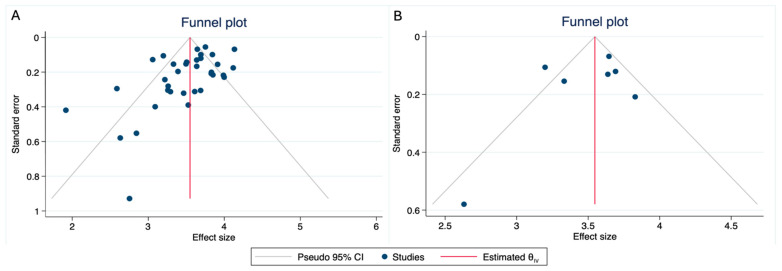
(**A**) Funnel plot for all studies included in systematic review and meta-analysis. (**B**) Funnel plot of studies with more than 100 sinonasal mucosal melanoma patients.

The funnel plot (Figure 3A) and the Egger’s regression test (*p* = 0.001) suggest a publication bias, with some studies reporting a worse prognosis compared with the more homogeneous group of studies. The studies identified as outliers were Cheng et al. [31], Lai et al. [39], Martin et al. [44], Wang T et al. [57], and Won et al. [15]. Among these, Cheng et al. and Martin et al. were two of the oldest series published, using a different staging classification. However, they did not present a higher proportion of patients with lymph node involvement or distant metastases. For the other studies, no significant differences were observed in TNM classification compared with the rest of cohorts (*p* = 0.593) or in treatment modalities (*p* = 0.157).

To evaluate the weight of smaller studies, we performed a funnel plot and 5-year survival analysis for studies that included more than 100 patients (7/35). The Egger’s test for these studies yielded a *p*-value of 0.286, although the funnel plot continued to show Won et al.’s study as an outlier, distancing itself from the rest of the more homogeneous studies (Figure 3B).

### 3.4. Patients’ Characteristics

The data are summarized in Table 1. A total of 35 studies were included, all of which were retrospective. Two studies (5.7%) were national databases, six (17.1%) were multicentric, twenty-six (74.3%) were unicentric, and one (2.8%) was a validation cohort. The study periods ranged from 1963 (Lund et al.) [41] to 2022 (Zhu et al. [60] and Durzynska et al. [33]).

The mean sample size was 2383 patients (SD 95.3). The largest patient cohort was reported by Lechner et al. (*n* = 505) [14], and the smallest by Göde et al. (*n* = 17) [36]. Fifteen studies (42.9%) were from Europe, six (17.1%) were from America, eleven (31.4%) were from Asia, two (5.7%) were from Australia, and one (2.9%) study combined European, United Kingdom and American populations.

There were 1192 (50%) females, 1085 (45.5%) males, and 106 (4.4%) whose sex was unknown. The age range was between 2 and 97 years, with a weighted mean age of 65.4 years (SD = 5.4). The lowest mean age was reported by Sun CZ et al. [52] at 47.6 years, and the highest was reported by Zebary et al. at 74 years [58].

Twenty-seven studies (77.1%) used the AJCC or UICC seventh or eighth edition staging for SNMM, three (8.6%) used the sixth edition, and three (8.6%) used the Ballantyne staging system. Among the first group using AJCC/UICC staging, a total of 1994 patients were included. Of these, 910 (45.6%) were classified as T3 at diagnosis, 529 (26.5%) as T4a, and 196 (9.8%) as T4b. Additionally, 157 patients (7.9%) were N(+) at diagnosis, while 1727 (86.6%) were N(−). Only 113 patients (5.7%) were M(+) at diagnosis, and 1604 (80.4%) were negative for distant metastasis.

Across all studies, the primary tumor site was the nasal cavity in 1507 cases (75%) and the paranasal sinuses in 541 cases (26.9%). Regarding primary treatment, 1781 patients (83.1%) underwent surgery, 63 (2.9%) received radiotherapy only, 19 (0.9%) received chemotherapy, 52 (2.4%) received high-dose proton beam therapy, 18 (0.8%) received chemoradiotherapy, 16 (0.7%) underwent debulking surgery, and 8 (0.4%) received immunotherapy. For adjuvant treatment, 960 patients (53.9%) received radiotherapy, 117 (6.6%) received chemotherapy, 84 (4.7%) received chemoradiotherapy, and 110 (6.2%) received immunotherapy.

**Table 1:** Description of included studies.

Author (Year)	Study Design	Period of Study	Sample Size and (Female n/%)	Age (Years) Mean (Range)	Follow-Up Months (Range)	T	N	M	Staging	Primary Tumor Site	5-Year OS (95% CI)	Primary Treatment	Adjuvant Treatment
Almutuawa (2020) [29]	R/U	1998–2015	20 (10/50)	71.3 (52–89)	16.8 (-) ^a^	T3 12 T4a 5 T4b 2	N/R	M0 19 M1 1	AJCC 7th edition	NC 11 PSs 8	40 (18.5–61.5) ^a^	S 20	RT 12
Bridger (2005) [30]	R/U	1970–1999	27 (12/44.4)	66 (23–84)	52	N/R	N/R	N/R	UICC 6th edition	NC 18 PSs 9	47 (28.2–65.8) ^a^	S 27	RT 14
Cheng (2007) [31]	R/U	1982–2002	23 (7/30.4)	68.2 (39–87)	(3–132)	N/R	N0 23 N1 0	M0 20 M1 3	Ballantyne staging	NC 7 PSs 9	15.7 (0.8–30.5) ^a^	S 20 RT 2 CT 1	RT 12 CT 1 CRT 2
Dréno (2017) [32]	R/U	1988–2015	44 (26/59.1)	71.2 (50–96)	50	T3 29 T4a 14 T4b 1	N0 42 N1 2	M0 39 M1 5	AJCC 7th edition	NC 30 PSs 15	33 (23.4–42.6) ^a^	S 42 RT 1	RT 22 CT 8 IT 14
Durzynska (2023) [33]	R/U	2011–2022	30 (17/56.7)	65 (34–90)	N/R	T3 18 T4a 10 T4b 2	N0 27 N1 3	M0 30 M1 0	UICC 8th edition	NC 16 PSs 14	32 (17–60)	N/R	N/R
Flukes (2021) [34]	R/U	1997–2018	100 (NR)	N/R	37	N/R	N/R	N/R	N/R	N/R	38 (28.5–47.5) ^a^	S 87	RT 79 IT 23
Fuji (2014) [35]	R/U	2006–2010	20 (8/40)	70.9 (55–81) ^a^	35 (6–77)	T3 9 T4a 8 T4b 3	N0 20 N1 0	N0 20 M1 0	UICC 7th edition	NC 12 PSs 8	54 (32.2–75.8) ^a^	PBT 20	CT 16
Göde (2017) [36]	R/U	2003–2014	17 (9/52.9)	65.4 (39–86)	59.8 (10–138)	T3 8 T4a 7 T4b 2	N0 13 N1 4	M0 17 M1 0	AJCC 7th edition	NC 7 PSs 3 Unk 7	61.4 (41.1–81.7) ^a^	S 17	RT 17
Hafström (2019) [37]	R/U	2001–2014	22 (12/54.5)	67.5 (51–83) ^a^	(5–138) ^a^	T3 3 T4b 3 Unk 16	N0 3 Unk 19	M0 22 M1 0	AJCC 7th edition	NC 14 PSs 8	50 (35.2–64.8) ^a^	S 9 RT 1 CRT 1 CRT+S 11	RT 4 CRT 1
Houette (2016) [38]	R/U	1998–2014	18 (11/61.1)	72 (54–94)	40	T3 12 T4a 4 T4b 2	N1 2	M1 3	AJCC 7th edition	NC 13 PSs 5	54.5 (31.5–77.5) ^a^	S 13 RT 2 CT 1 DS 2	RT 15
Lai (2020) [39]	R/U	2000–2016	92 (40/43.5)	66.5 (28–89)	27 (1–133)	T3 46 T4a 32 T4b 11	N0 90 N1 2	M0 92 M1 0	AJCC 7th edition	N/R	13.3 (6.4–20.2) ^a^	S 92	RT 23 CT 3 CRT 22
Lechner (2023) [14]	R/M	1999–2021	505 (271/53.7)	60.5 (15–93) ^a^	21.3	T3 239 T4a 120 T4b 39 Unk 107	N0 447 N1 43 Unk 15	M0 329 M1 20 Unk 156	AJCC 7th edition	NC 411 PSs 199	38.3 (33.2–43.4)	S 431 RT 4 CT 6 CRT 6	RT 192 CT 19 CRT 23 IT 22
Ledderose (2023) [40]	R/U	2002–2015	27 (12/44.4)	68 (33–94)	33 (1.5–164.5)	T3 14 T4a 9 T4b 4	N0 27 N1 0	M0 27 M1 0	AJCC 7th edition	N/R	33.3 (24.2–42.4) ^a^	S 27	RT 27
Lund (2012) [41]	R/U	1963–2010	115 (64/55.7)	65.9 (15–91)	37.5 (2–360)	N/R	N0 101 N1 10 Unk 4	M0 115 M1 0	N/R	NC 90 PSs 19 Unk 6	28 (19.8–36.2) ^a^	S 115	RT 41 CT 5 CRT 10
Lundberg (2019) [42]	R/U	1983–2016	58 (31/53.4)	71.9 (43–95) ^a^	16 (1–229)	T3 29 T4a 16 T4b 8 Unk 5	N0 49 N1 4 Unk 5	M0 54 M1 4	UICC 7th edition	NC 45 PSs 13	25 (13.9–36.1) ^a^	S 43 RT 1 RT+S 1 CT 2 DS 11	RT 17
Manton (2019) [43]	R/U	2009–2017	31 (22/71)	71 (52–85)	38.5	T3 19 T4a 1 T4b 7 Unk 4	N0 22 Unk 9	M0 31 M1 0	AJCC 7th edition	NC 23 PSs 8	46.7 (37.7–55.7) ^a^	S 31	RT 26
Martin (2004) [44]	R/U	1991–2002	20 (12/60)	72.3 (45–91) ^a^	79 (7–128)	Unk 20	N0 20 N1 0	M0 20 M1 0	UICC 6th edition	NC 8 PSs 3 NC + PSs 9	17.2 (4.3–37.5) ^a^	S 18 RT 2	RT 15 CT 1
Meerwein (2019) [45]	R/U	1992–2018	34 (19/55.9)	71.2 (IQR 63–78)	16.5	T3 18 T4a 7 T4b 9	N0 32 N1 2	M0 32 M1 2	AJCC 7th edition	NC 29 PSs 5	62.4 (54.1–70.7) ^a^	S 30 RT 3	RT 25
Michel (2014) [46]	R/U	1995–2010	35 (20/57.1)	64.8 (36–84)	49 (4–255)	T3 4 T4a 30 T4b 1	N0 35 N1 0	M0 33 M1 2	AJCC 7th edition	NC 27 PSs 7 NP 1	26.9 (12.2–41.6) ^a^	S 30 CT 3 CRT 2	RT 11 IT 2
Miglani (2017) [47]	R/ U	1999–2015	22 (14/63.6)	72 (48–92)	25 (1.7–172.9)	T3 9 T4a 10 T4b 3	N0 19 N1 3	M0 19 M1 3	AJCC 7th edition	NC 18 PSs 4	37 (16.8–57.2) ^a^	S 22	RT 13 CRT 3
Mochel (2014) [48]	R/U	1990–2012	32 (13/40.6)	65.3 (30–90) ^a^	16 (5–211)	T3 12 T4a 8 T4b 5 Unk 7	N0 29 N1 3	M0 32 M1 0	AJCC 7th edition	NC 11 PSs 4 Unk 17	22 (7.6–36.4) ^a^	N/R	N/R
Narasimhan (2009) [49]	R/U	1995–2007	18 (10/55.6)	62.5 (31–85) ^a^	N/R	N/R	N0 7 N1 11	M0 12 M1 6	AJCC 6th edition	NC 9 PSs 12	34 (12.1–55.9) ^a^	N/R	RT 4 CT 2 CRT 6 IT 8
Rojas- Lechuga (2022) [50]	R/M	1984–2020	50 (26/52)	70.4 (40–95)	39.6	T3 22 T4a 19 T4b 9	N0 43 N1 7	M0 44 M1 6	AJCC 8th edition	NC 24 PSs 26	26.1 (13.5–40.6)	S 41	RT 20
Scherzad (2023) [51]	R/U	1999–2020	38 (22/57.9)	N/R	33 (3–115)	T3 19 T4a 9 T4b 10	N0 33 N1 5	M0 30 M1 4 Unk 4	UICC 8th edition	NC 32 PSs 6	26 (12.1–39.9) ^a^	S 21 DS+RT 8 DS 3 IT 3 CT+IT 1	RT 9 RT+IT 1
Scheurleer (2024) [9]	R/D	2001–2021	320 (174/54.4)	73.1 (IQR 65–81.5) ^a^	20.2	T3 95 T4a 54 T4b 26 Unk 20	N0 248 N1 30 Unk 42	M0 287 M1 33	UICC 7th and 8th edition	NC 262 PSs 51 Unk 7	24.5 (19.6–29.7)	S 245 RT 32 CRT 9	RT 176 CRT 6
Sun CZ (2013) [52]	R/U	1976–2005	68 (21/30.9)	47.6 (2–79) ^a^	24 (1–264)	T3 36 T4b 4 Unk 28	N0 63 N1 5	M0 64 M1 4	AJCC 7th edition	NC 55 PSs 13	29.7 (18.8–40.6) ^a^	S 51 RT 4 CT 6 CRT+IT 2 CT+IT 2	RT 13 RT+IT 1 CT 9 CT+IT 10
Sun S (2023) [53]	R/D	1990–2020	107 (52/48.6)	57.2 (IQR 49–64) ^a^	118 (3–273)	T3 44 T4a 53 T4b 9 Unk 1	N0 102 N1 5	M0 107 M1 0	AJCC 7th edition	NC 96 PSs 11	40.1 (30.8–49.4) ^a^	S 103 RT 4	RT 63
Thariat (2011) [54]	R/M	1991–2006	25 (18/72)	70.4 (45–91) ^a^	37 (1–181)	N/R	N0 24 N1 1	M0 25 M1 0	N/R	NC 20 PSs 5	38 (26–50)	S 23 RT 2	RT 9 RT + IT 1
Tsushima (2023) [55]	R/U	2002–2021	30 (12/40)	67.9 (45–83) ^a^	26.4 (6–103.2)	T3 22 T4a 8 T4b 0	N0 28 N1 2	M0 30 M1 0	UICC 7th edition	NC 28 PSs 2	42.6 (38–47.2) ^a^	S 28 RT 2	RT 28
Wang L (2022) [56]	R/M	2007–2018	117 (60/51.3)	60 (37–83) ^a^	21 (0–77)	T3 87 T4a 14 T4b 16	N0 112 N1 5	N/R	AJCC 7th edition	NC 84 PSs 23 Unk 10	46 (28.2–63.8) ^a^	N/R	N/R
Wang T (2020) [57]	R/U	2008–2017	36 (17/47.2)	67.4 (N/R)	22 (4–96)	T3 8 T4a 23 T4b 5	N0 27 N1 9	M0 32 M1 4	AJCC 7th edition	NC 23 PSs 13	6.8 (2.2–11.4) ^a^	S 33 RT 3	RT 13
Won (2015) [15]	R/M	1994–2013	155 (74/47.7)	63.3 (28–92)	40.9 (12–200)	T3 71 T4a 54 T4b 9 Unk 21	N0 139 N1 16	M0 141 M1 14	AJCC 7th edition	N/R	13.9 (2.6–25.2 ^a^)	S 133	RT 43 CT/IT 17 CRT/IT 11
Zébary (2013) [58]	R/U	1986–2011	61 (35/57.4)	74 (52–97)	N/R	N/R	N0 52 N1 Unk 3	M0 53 M1 1 Unk 2	Ballantyne staging	NC 34 PSs 22	40.1 (32.4–47.8) ^a^	N/R	N/R
Zenda (2016) [59]	R/M	2008–2011	32 (20/62.5)	67.6 (36–89) ^a^	36.2	T3 11 T4 21	N0 32 N1 0	M0 32 M1 0	UICC 7th edition	NC 28 PSs 4	21.3 (16.1–26.5) ^a^	PBT 32	N/R
Zhu (2024) [60]	R/V	2000–2022	34 (21/61.8)	N/R	30.5	T3 14 T4a 14 T4b 6	N0 29 N1 5	M0 26 M1 8	AJCC 7th edition	NC 22 PSs 12	46.1 (28.8–63.4) ^a^	S 29	RT 17 CT 24

Abbreviations: R, retrospective; U, unicentric; M, multicentric; D, national database; V, validation cohort; NC, nasal cavity; PSs, paranasal sinuses; S, surgery, RT, radiotherapy; CT, chemotherapy; CRT, chemoradiotherapy; IT, immunotherapy; PBT, proton beam therapy; Unk, unknown; DS, debulking surgery; NP, nasopharynx; IQR, interquartile range. ^a^ Estimated data extracted from numbers or graphs.

### 3.5. Overall Survival

From the 35 included studies, the cumulative 5-year OS was 34.8 (95% CI = 30.6–39.5, τ2 = 0.1). Overall, results were heterogeneous among studies, with an I^2^ = 82.0% (Figure S1). The cumulative 5-year OS for studies with more than 100 patients was 33.83 (95% CI = 28.1–40.8, τ2 = 0.04, I^2^ = 70.8%) (Figure S2).

**Figure 4:**
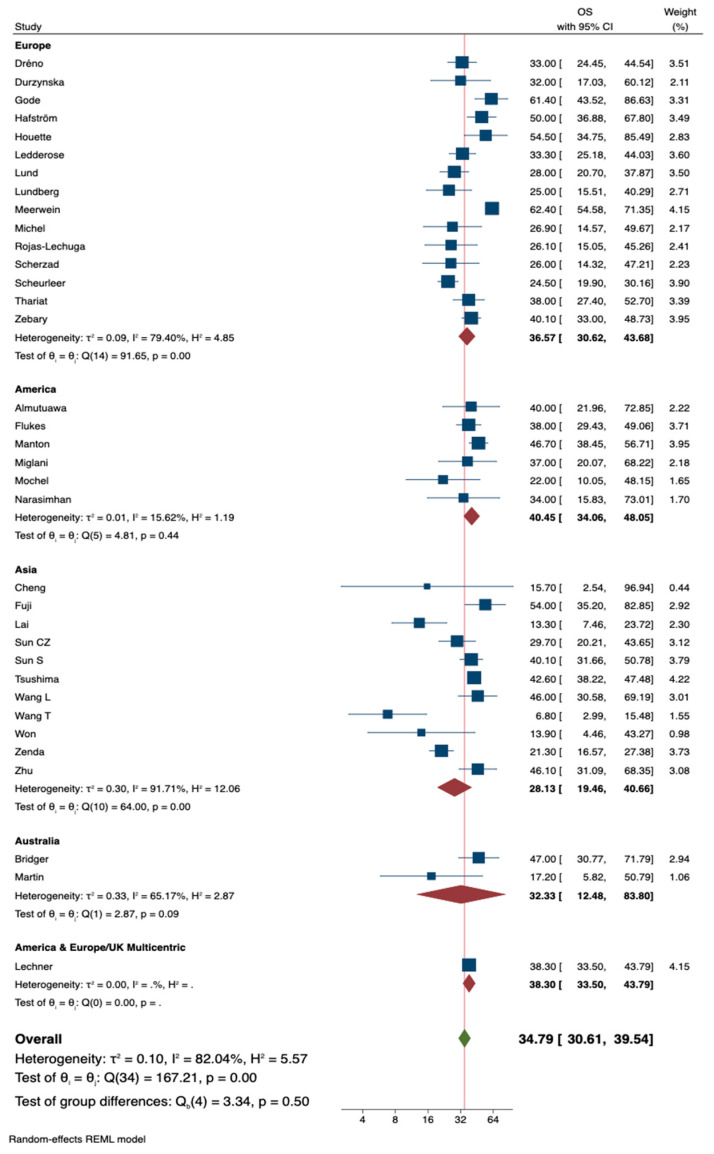
Forest plot representing the overall survival (OS) by geographical location [9,14,15,29,30,31,32,33,34,35,36,37,38,39,40,41,42,43,44,45,46,47,48,49,50,51,52,53,54,55,56,57,58,59,60].

In the sub-analysis by geographic area, better survival outcomes were observed in the American group, with a 5-year OS of 40.5 (95% CI = 34.1–48.1, τ2 = 0.01, I^2^ = 15.6%), while the worst outcomes were seen in the Asian group, with a 5-year OS of 28.1 (95% CI = 19.5–40.7, τ2 = 0.3, I^2^ = 91.7%) (Figure 4).

In the sub-analysis by T stage, the cumulative 5-year OS for T3 (n = 461) was 41.8 (95% CI = 32–54.7, τ2 = 0.10, I^2^ = 92.9%); for T4a (n = 176) it was 23.2 (95% CI = 14.6–37, τ2 = 0.14, I^2^ = 71%), and for T4b (n = 80) it was 0.5 (95% CI = 0.01–37.4, τ2 = 25, I^2^ = 99.7%) (Figure S3).

### 3.6. Histopathologic, Genetic Mutations and Biomarkers in SNMM

Histopathological analysis revealed melanin presence in 61.9% of cases (284/459). Among the samples, 76.5% (52/68) were positive for *S-100*, 94.1% (64/68) for *HMB-45*, 83.6% (56/67) for *Melan-A*, 100% (29/29) for *SOX-10*, and 76% (19/25) for *PRAME* (preferentially expressed antigen in melanoma). Mitosis showed significant heterogeneity in measurement methods, with some studies reporting in mm^2^ and others in 10 HPF, complicating comparisons across series.

The positive rate for the *BRAF* pV600 mutation by qPCR was 2.4% (3/125) and 2.3% (1/43) by immunohistochemistry (IHC). *c-KIT* positivity was found in 36.4% of samples (12/33) by IHC and in 5% of samples by *KIT* qPCR (5/101). In next-generation sequencing (NGS), 17.7% (17/96) were found to have *NRAS* mutations, while 10.5% (2/19) had *KRAS* mutations.

### 3.7. Incidence

**Figure 5:**
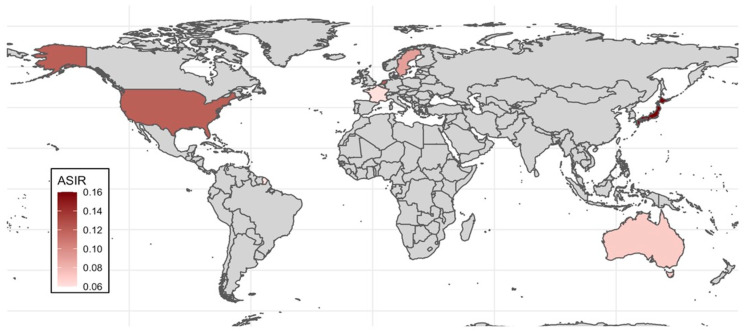
Age-standardized incidence rate (ASIR) for sinonasal mucosal melanoma by country.

Only three countries (Australia [61], Sweden [2], and the Netherlands [9]) have published the ASIR in SNMM. The United States reported ASIR for head and neck mucosal melanomas (0.12 per 100,000 person/year) [62] or reported specific rates divided by nasal cavity and paranasal sinuses, with 0.03 and 0.02 per 100,000 person/year, respectively [8]. Japan [12] and France [63] described their crude incidence rates, but these were not age-standardized and should be viewed cautiously, as they are not directly comparable with the rest of the populations (Figure 5).

The lowest ASIR was reported in Australia at 0.085 per 100,000 person/year, and the highest was reported in the Netherlands at 0.12 per 100,000 person/year. In addition, lower incidence rates were reported for males compared with females in all countries (Table 2).

An upward incidence trend has been observed in Sweden (1960–2000) [2], Australia (1985–2009) [61], and the United States (1987–2009) [62], whereas the Netherlands has described stable incidence rates (2001–2021) with an annual percentage change of 0.01% [9].

## 4. Discussion

Sinonasal tumors, although traditionally uncommon in head and neck tumors, have gained greater understanding and recognition due to their high morbidity. While previous research has mainly consisted of single-institution, retrospective reports, recent efforts have focused on multi-institutional studies, improving evidence quality. This progress has been made possible by collaboration and knowledge sharing among multidisciplinary specialists involved in managing these pathologies [16].

Despite these advances, SNMM remains one of the most challenging sinonasal tumors, with a significant need for further understanding. In the series of 940 patients with sinonasal tumors by Arosio et al., SNMM was identified as the most aggressive histologic subtype with the poorest prognosis [64]. SNMM presents high rates of locoregional recurrence and distant metastasis that significantly impact patient survival [21].

We have confirmed these findings of poor prognosis with a 5-year survival rate of 34.8% in a cumulative cohort of 2383 SNMM patients. Survival rates were 41.8% for T3, 23.2% for T4a, and close to zero when it affected neurovascular structures (T4b). The most common primary site was the nasal cavity (75%), while 26.9% of cases occurred in the paranasal sinuses. These findings align with a large series suggesting that increased infiltration depth (e.g., cartilage or bony structures of the paranasal sinus region) significantly raises the risk of systemic dissemination [14,64].

We found a low rate of lymph node metastasis (7.9%) and distant metastasis (5.7%) at diagnosis, which emphasizes the importance of achieving good locoregional control to prevent systemic dissemination. It is important to note that several articles excluded patients who presented with metastatic disease at diagnosis [36,39,40,41,43,44,48,53,54,55,59]. Additionally, Ledderose et al. excluded patients with extensive malignant infiltration of critical structures, such as the brain, skull base, carotid artery, or prevertebral space [40], and Manton et al. excluded patients with unresectable disease [43]. This exclusion criterion is significant as it may have led to higher reported OS rates in these studies.

In our meta-analysis, most patients (83.3%) underwent surgery, in line with NCCN guidelines [65], and 53.9% received adjuvant radiotherapy. Only 6.2% of patients received immunotherapy, which consisted mainly of immune checkpoint inhibitors (ICIs), although in several articles, they also described the use of bacillus Calmette–Guerin (BCG) vaccine, interleukin (IL)-2, or interferon (IFN)-ɑ-2b [14,15,32,46,49,51,52,54]. A recent meta-analysis by Tang et al. on immunotherapy treatment for SNMM described 117 patients treated with adjuvant or salvage ICI immunotherapy, with a positive response rate for tumor volume reduction or resolution of 40.2% (95% CI: 36.8–43.6). They also reported a 5-year OS after adjuvant immunotherapy treatment of 42.6% (95% CI: 39.4–45.8), which is slightly higher than our global 5-year OS of 34.8 (95% CI = 30.6–39.5), indicating a promising trend toward survival improvement with ICI treatment [25]. The role of neoadjuvant immunotherapy, however, remains to be elucidated.

The current literature demonstrates significant heterogeneity in reporting survival outcomes, highlighting the need for greater standardization in scientific publications. While the TNM classification of head and neck mucosal melanomas has been an important step forward on this path, it still falls short in accurately stratifying patients within the T3 and T4a categories. This has led to an ongoing debate about whether additional variables should be incorporated into future TNM editions. Lechner et al. have suggested incorporating sinus involvement for T3 tumors [14]. Other authors have also described worse prognosis biomarkers, such as tumor infiltrating lymphocytes (TILs) [25,66,67,68], *PRAME* [33], and mitotic rates [43,50,69,70]. Therefore, further efforts are needed to better discern the survival and molecular profiles of patients with SNMM.

This meta-analysis is the first to compare the survival of SNMM across different geographical regions, showing lower heterogeneity and better survival outcomes in America (40.5%), followed by Europe (36.6%), Australia (32.3%), and Asia (28.1%). Further studies are required to determine whether the poorer OS observed in Asia is related to ethnic factors, differences in accessibility to healthcare, or whether a distinct molecular profile contributes to the worse behavior of tumors in this population.

The ASIR for SNMM is between 0.072 and 0.149 per 100,000 person/year, with a slightly higher incidence described in women than in men [2,9,61]. An upward trend in incidence for both sexes has been described in Sweden [2], Australia [61], and the United States [62], similar to the trend seen in cutaneous melanoma [71]. However, SNMM does not appear to be driven by ultraviolet radiation exposure [7]. The etiologic factors driving tumorigenesis of SNMM have not been established and require further investigation. Understanding whether this increasing trend is exclusively due to population growth, better diagnostic tools, or is influenced by greater exposure to risk factors is essential for managing this malignancy more effectively.

### Strengths and Limitations

The main limitation is the quality of the available evidence, mainly case series and retrospective single- or multicenter cohort studies. Nevertheless, it is essential to note that for rare diseases, case series provide the most pertinent source of data and are more reliable than registry databases or other sources. Our meta-analysis showed significant heterogeneity among the studies in terms of OS, which may be attributed to differences in patient characteristics, different times and treatment modalities, misdiagnosis, different access to healthcare, or possible distinctive genetic profiles. Additionally, many studies lacked molecular data, limiting our ability to perform detailed sub-analyses to understand whether this heterogeneity could be attributed to molecular differences. The results should be interpreted with caution as, in a random-effects model, it is possible to overweigh small studies. We have considered this limitation and have performed a sub-analysis exclusively with the large series (>100 patients), which does not significantly vary the 5-year OS (difference of 0.95, *p*-value = 0.317). The publication bias identified with studies with worse prognosis must also be taken into consideration, which must be evaluated in the future if they correspond to the clinicopathological characteristics of the tumor or differences in access to the health system, such as if they were treated in a referral cancer center, among others. Another limitation is the non-inclusion of literature in Asian languages that may contribute to this rare subtype of melanoma. In addition, the underrepresentation of data from certain countries may weaken the reliability of generalizing these findings on a global scale. Despite these limitations, our analysis provided valuable insight into the prognosis of this rare malignancy.

## 5. Conclusions

This study presents one of the largest cohort analysis to date on SNMM, a rare and aggressive form of cancer with historically poor survival outcomes. Despite geographic differences in survival rates, the overall 5-year survival remains low. This meta-analysis provides a comprehensive global vision of the disease and encourages further research into the complex nature of this challenging tumor, improves understanding, and supports the development of tailored treatment strategies.

## Figures and Tables

**Table 2:** Age-standardized incidence rate of sinonasal mucosal melanoma.

Author	Year	Country	Sex	ASIR
Scheurleer [9]	2021	Netherlands	Female	0.14 ^a^
			Male	0.1 ^a^
Youseff [61]	2005–2009	Australia	Female	0.09 ^a^
			Male	0.08 ^a^
Jangard [2]	1995–2000	Sweden	Female	0.11 ^a^
			Male	0.07 ^a^
Marcus [62]	2009	United States	Both	0.12 *
Beaudoux [63]	2004–2014	France	Both	0.06 ^ab^
Tomizuka [12]	2011–2013	Japan	Both	0.16 ^b^

^a^ Estimated data extracted from numbers or graphs. ^b^ Crude incidence rates. * Head and neck mucosal melanoma incidence rate.

## Data Availability

No new data were created or analyzed in this study.

## Supplementary Materials

The following supporting information can be downloaded at: https://www.mdpi.com/article/10.3390/jpm14121120/s1, File S1: articles excluded with their reason [2,21,22,23,55,66,67,68,69,70,72,73,74,75,76,77,78,79,80,81,82,83,84,85,86,87,88,89,90,91,92,93,94,95,96,97,98,99,100,101,102,103,104,105,106,107,108,109,110,111,112,113,114,115,116,117,118,119,120,121,122,123,124,125,126]; File S2: GRADE assessment; Figure S1: Forest plot representing the overall survival (OS) [9,14,15,29,30,31,32,33,34,35,36,37,38,39,40,41,42,43,44,45,46,47,48,49,50,51,52,53,54,55,56,57,58,59,60]; Figure S2: Forest plot representing the overall survival (OS) in studies over 100 sinonasal mucosal melanoma patients [9,14,15,34,41,53,56]; Figure S3: Forest plot representing the overall survival (OS) by tumor staging; and Table S1: List of excluded articles and reasons [14,32,39,50,53,55,56,57].
