# Supplementary material for: Survival Outcomes in Sinonasal Mucosal Melanoma: Systematic Review and Meta-Analysis
Source: J Pers Med. 2024 Nov 26;14(12):1120. doi: 10.3390/jpm14121120 (PMC11678336; doi:10.3390/jpm14121120)
Supplement: Supplementary file 1 [file jpm-14-01120-s001.zip › SUPPLEMENTARY FIGURE LEGENDS.pdf]

SUPPLEMENTARY FIGURE LEGENDS

Figure S1. Forest Plot representing the Overall Survival (OS).

Figure S2. Forest Plot representing the Overall Survival (OS) in studies over 100 Sinonasal Mucosal Melanoma patients.

Figure S3. Forest Plot representing the Overall Survival (OS) by Tumor staging
