# Supplementary material for: Survival Outcomes in Sinonasal Mucosal Melanoma: Systematic Review and Meta-Analysis
Source: J Pers Med. 2024 Nov 26;14(12):1120. doi: 10.3390/jpm14121120 (PMC11678336; doi:10.3390/jpm14121120)
Supplement: Supplementary file 1 [file jpm-14-01120-s001.zip › Supplementary File S2.pdf]

Supplementary Table S1: GRADE Assessment

**Outcomes**	**Pooled outcomes (95% CI)**		**No. of patients (no. of included studies)**	**Statistical heterogeneity**	**Quality of evidence (GRADE)**
Melanoma overall survival	34.79 (30.61, 39.54)		2383 (35 studies)	I^2 = 82.04% (*P* = <0.00001)	⊕⊖⊖⊖[b,c]
Melanoma overall survival (studies with ≥100 patients)	33.83 (28.06, 40.80)		1419 (7 studies)	I^2 = 70.77% (*P* = <0.00001)	⊕⊖⊖⊖[b]
Melanoma overall survival (geographic area)	Europe	36.57 (30.62, 43.68)	894 (15 studies)	I^2 = 79.40% (*P* = <0.00001)	⊕⊖⊖⊖[b]
	America	40.45 (34.06, 48.05)	223 (6 studies)	I^2 = 15.62% (*P* = 0.44)	⊕⊖⊖⊖
	Asia	28.13 (19.46, 40.66)	714 (11 studies)	I^2 = 91.71% (*P* = <0.00001)	⊕⊖⊖⊖[b#]
	Australia	32.33 (12.48, 83.80)	47 (2 studies)	I^2 = 65.17% (*P* = 0.09)	⊕⊖⊖⊖[a,b#]
Melanoma overall survival (T stage)	T3	41.82 (32.00, 54.66)	451 (7 studies)	I^2 = 92.87% (*P* = <0.00001)	⊕⊖⊖⊖ [b]
	T4a	23.21 (14.55, 37.00)	176 (4 studies)	I^2 = 70.95% (*P* = 0.01)	⊕⊖⊖⊖ [b]
	T4b	0.46 (0.01, 37.37)	80 (5 studies)	I^2 = 99.66% (*P* = <0.00001)	⊕⊖⊖⊖[a,b*]

[a] Downgraded by one level for imprecision (total number of studies is less than 3 and/or wide confidence intervals).
[b] Downgraded by one level for inconsistency (statistical heterogeneity p<0.05, large variation in effect[#], confidence intervals do not overlap*)
[c] Downgraded by one level for the possibility of publication bias.
No studies were downgraded by indirectness of evidence.
No studies were downgraded for risk of bias by JBI.
