# Supplementary figures and images for: Survival Outcomes in Sinonasal Mucosal Melanoma: Systematic Review and Meta-Analysis
Source: J Pers Med. 2024 Nov 26;14(12):1120. doi: 10.3390/jpm14121120 (PMC11678336; doi:10.3390/jpm14121120)

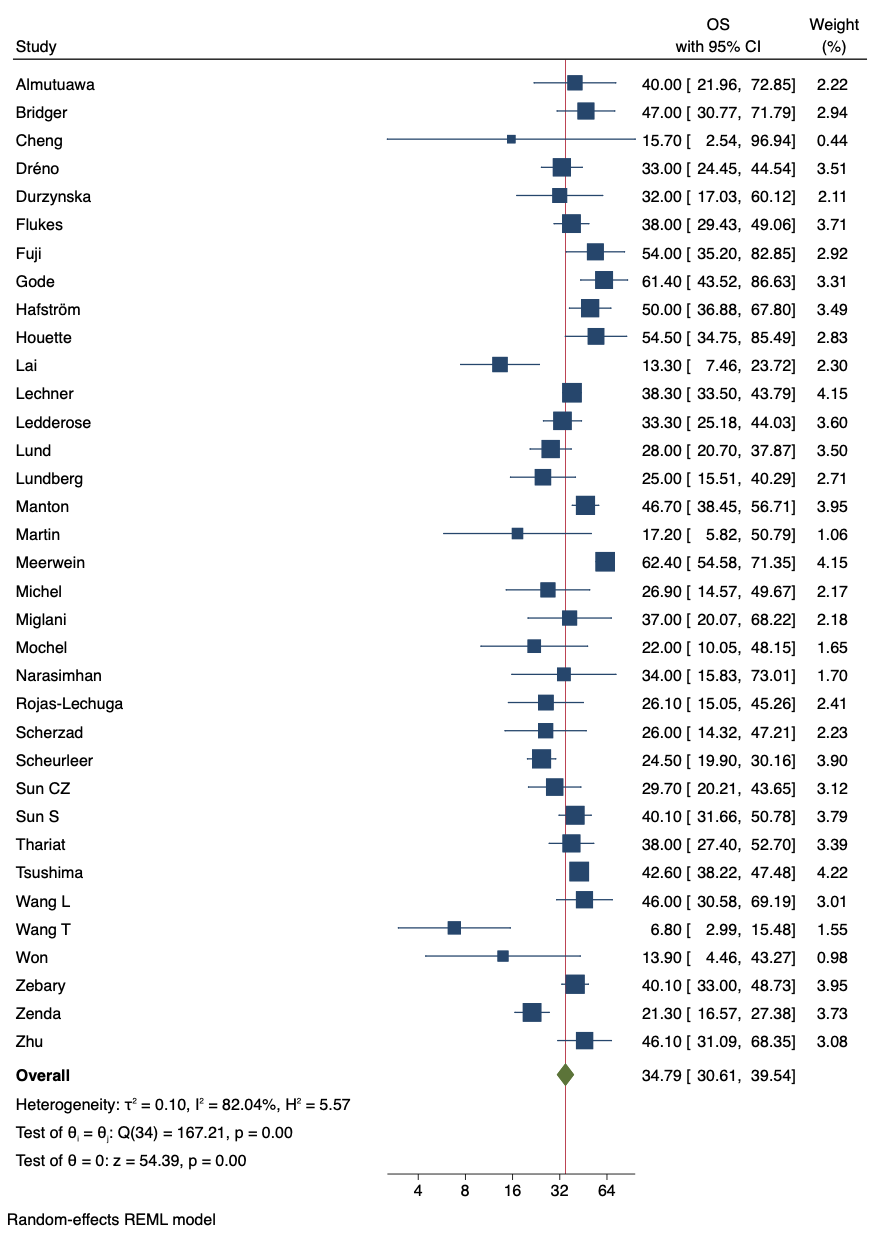

Supplement: Supplementary file 1 [file jpm-14-01120-s001.zip › FigureS1.png]

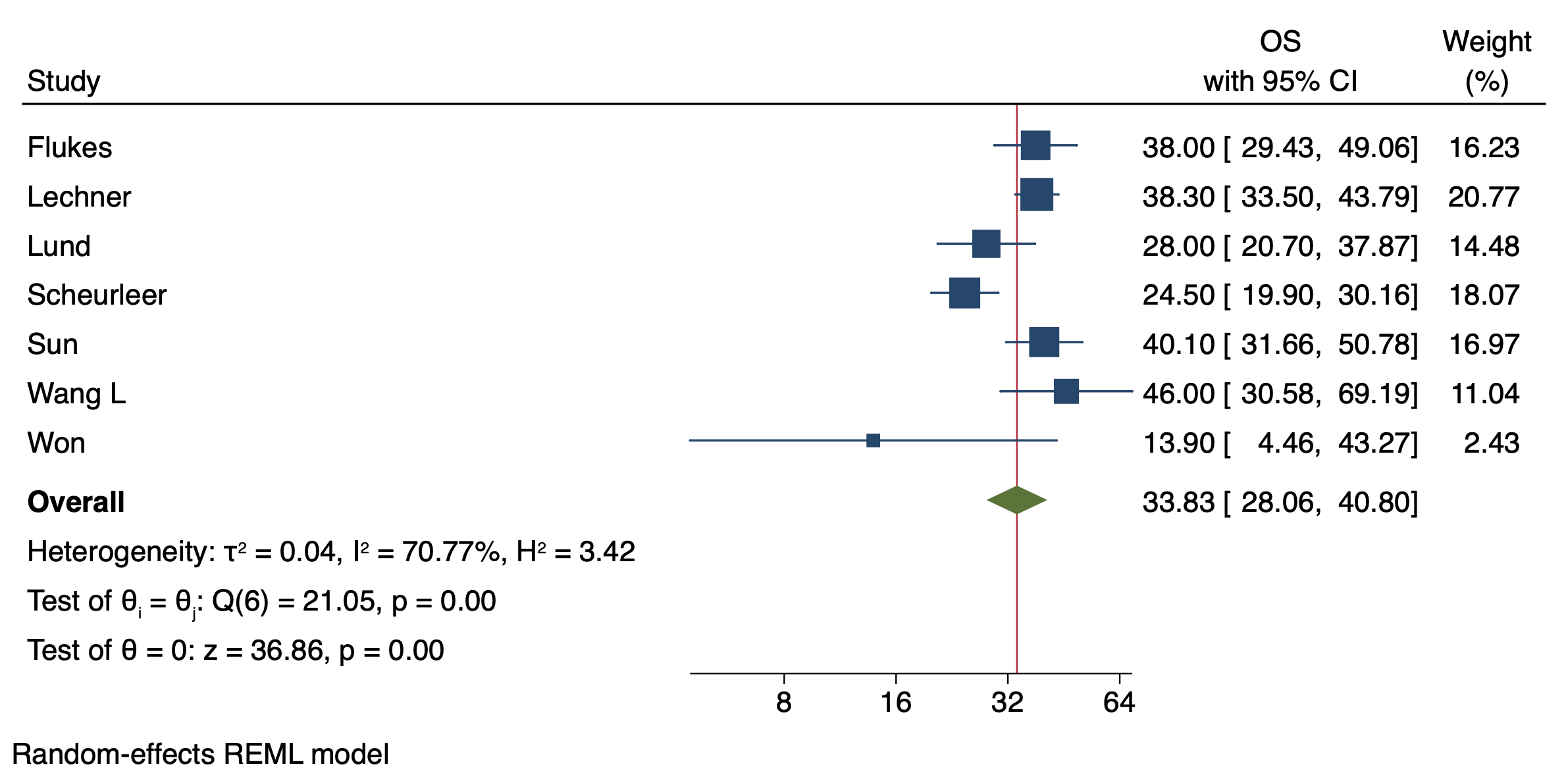

Supplement: Supplementary file 1 [file jpm-14-01120-s001.zip › FigureS2.png]

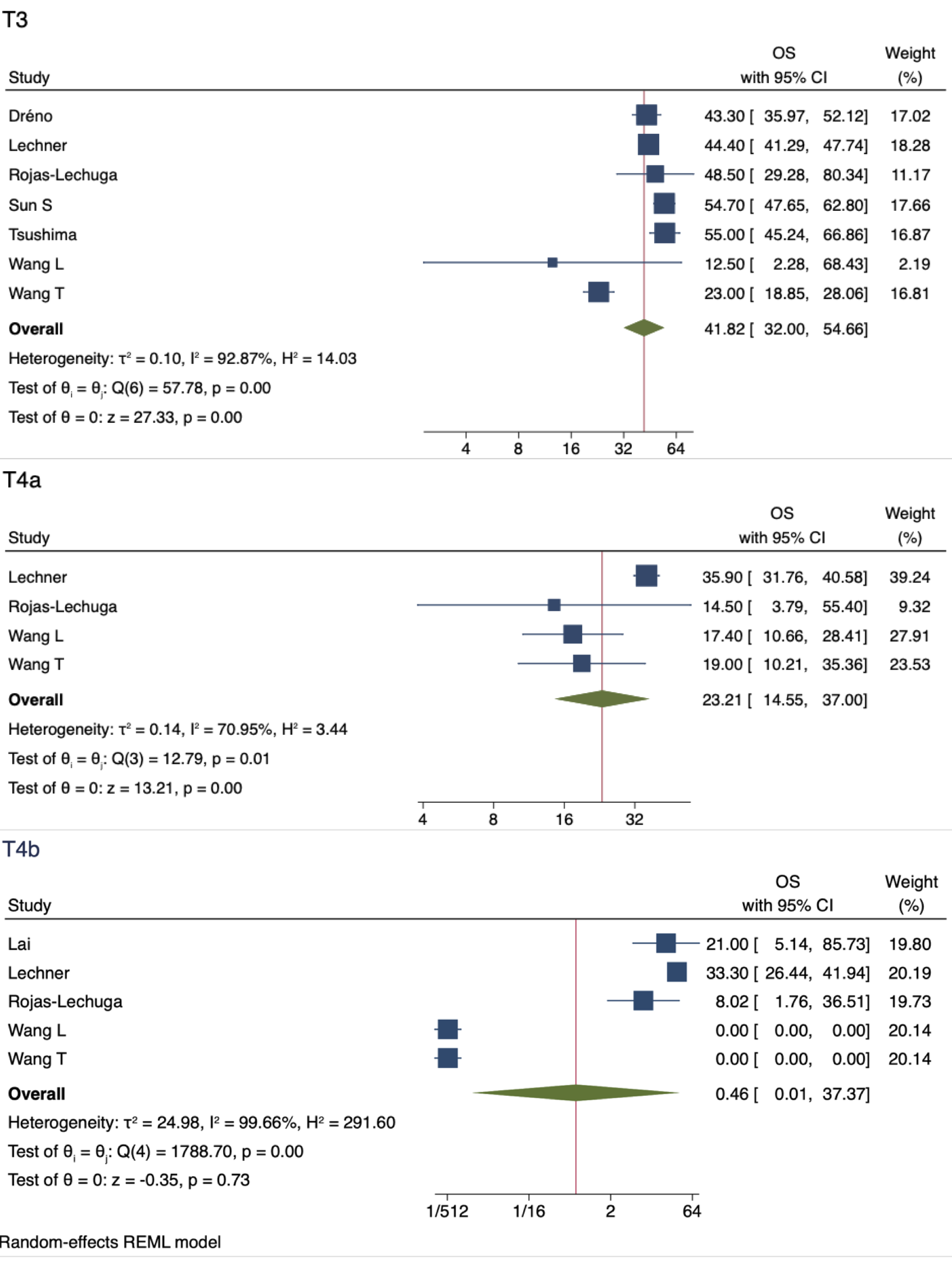

Supplement: Supplementary file 1 [file jpm-14-01120-s001.zip › FigureS3.png]
